# Phase-transition-induced jumping, bending, and wriggling of single crystal nanofibers of coronene

**DOI:** 10.1038/s41598-021-82703-5

**Published:** 2021-02-04

**Authors:** Ken Takazawa, Jun-ichi Inoue, Kazutaka Mitsuishi, Yukihiro Yoshida, Hideo Kishida, Paul Tinnemans, Hans Engelkamp, Peter C. M. Christianen

**Affiliations:** 1 Center for Green Research on Energy and Environmental Materials, National Institute for Materials Science, Tsukuba, Ibaraki 305-0003 Japan; 2 MANA, National Institute for Materials Science, Tsukuba, Ibaraki 305-0044 Japan; 3 Research Center for Advanced Measurement and Characterization, National Institute for Materials Science, Tsukuba, Ibaraki 305-0047 Japan; 4 Division of Chemistry, Graduate School of Science, Kyoto University, Kitashirakawa-Oiwakecho, Sakyo-ku, Kyoto, 606-8502 Japan; 5 Faculty of Agriculture, Meijo University, Tempaku-ku, Nagoya, 468-8502 Japan; 6 Department of Applied Physics, Nagoya University, Chikusa-ku, Nagoya 464-8603 Japan; 7 Department of Solid State Chemistry, Radboud University, 6500 GL Nijmegen, The Netherlands; 8 High Field Magnet Laboratory (HFML-EMFL), Radboud University, 6525 ED Nijmegen, The Netherlands

**Keywords:** Nanowires, Chemical physics

## Abstract

For decades, it has been reported that some organic crystals suddenly crack, break, or jump when they are heated from room temperature. Recently, such crystals have been intensively studied both in fundamental science and for high-speed mechanical device applications. According to these studies, the sudden crystal motions have been attributed to structural phase transitions induced by heating. Stress created by the phase transition is released through the sudden and rapid motion of the crystals. Here we report that single crystal nanofibers of coronene exhibit a new type of ultrafast motion when they are cooled from room temperature and subsequently heated to room temperature. The nanofibers make centimeter-scale jumps accompanied by surprisingly unique behaviors such as sharp bending and wriggling. We found that the motions are caused by a significantly fast structural phase transition between two polymorphs of coronene. A theoretical investigation revealed that the sudden force generated by the phase transition together with the nanoscale dimensions and elastic properties create *dynamical instability* in the nanofibers that results in the motions. Our finding demonstrates the novel mechanism that leads to ultrafast, large deformation of organic crystals.

## Introduction

Since the 1980s, several groups have reported observations of jumping crystals or thermosalient crystals. In early studies, crystals of organic molecules, including organometallics, were serendipitously found to suddenly crack, break, or jump when they were heated above room temperature^1–8^. In addition to visual observation of the crystal motions, X-ray diffraction and thermodynamic measurements were performed by heating the crystals. These measurements showed that the crystal motions are related to temperature-induced structural phase transitions between polymorphs of the crystals. However, these early studies were rather sporadic and focused not on the underlying mechanism of the sudden motions but on the properties of the specific crystals.

In recent years, the thermosalient effect, as well as the photosalient effect, in which light irradiation induces sudden crystal motions, have attracted increasing interest from both fundamental science and device application perspectives. Naumov and coworkers have intensively studied thermosalient crystals using crystallographic, spectroscopic, and thermodynamic methods and discussed the common characteristics of the phase transitions that cause the sudden crystal motions^9–18^. They also performed kinematic analyses of the crystal motions by recording them with a high-speed camera. These studies have advanced the understanding of the mechanisms behind the sudden crystal motions and stimulated a number of related studies, including those aimed at exploring applications in high-speed mechanical devices and heat-motion conversion devices^19–23^.

Here we report that nanoscale organic crystals exhibit a novel type of ultrafast motion owing to temperature-induced structural phase transitions. We observed that single crystal nanofibers of coronene on a substrate jumped over a centimeter-scale distance at an initial velocity of up to ~ 20 m/s, which is a few orders of magnitude faster than that of reported thermosalient crystals. Most thermosalient effects known so far take place by heating the crystals above room temperature and a few have been reported to occur below room temperature^24^. The jumps of our nanofibers occur as they are cooled from room temperature, and the cooled nanofibers jump again when subsequently heated to room temperature. We investigated the jump of the nanofibers with crystallographic and microscopy methods and found that it is attributed to a significantly fast structural phase transition between two polymorphs of coronene. Moreover, ultrahigh-speed camera recording revealed surprising behaviors of the nanofibers at the moment of jumping, i.e., sharp bending and wriggling motions. The theoretical investigation revealed that the abrupt force generated by the phase transition together with the nanoscale dimensions and elastic properties create a *dynamical instability* in the nanofibers and this instability leads to the motion. Our findings demonstrate the novel mechanism for ultrafast large deformation of organic crystals, which provide a new insight into the mechanical properties of organic crystals and have potential applications in nanoscale mechanical devices.

## Results and discussion

**Figure 1:**
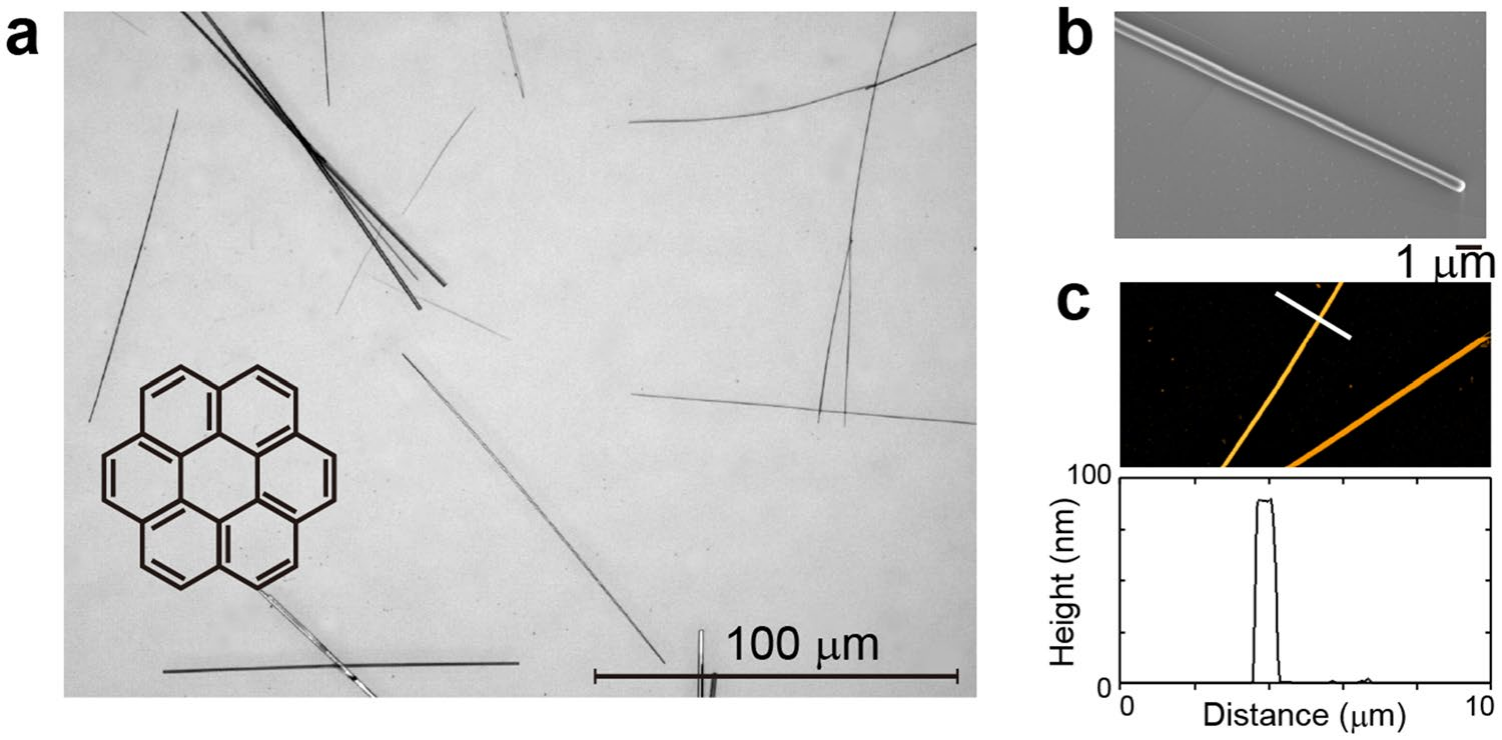
Morphology of coronene nanofibers. **(a)** Optical micrograph of coronene nanofibers on substrate. Inset: chemical structure of coronene. (**b)** SEM image of a coronene nanofiber. (**c)** Upper panel: AFM image of coronene nanofibers. Lower panel: cross section of the nanofiber along the white line in the upper panel.

Coronene, or [6]circulene, is a polyaromatic hydrocarbon molecule consisting of six benzene rings (Fig. 1a, inset). Single crystal nanofibers of coronene with lengths of 20–200 µm were prepared by solution-phase self-assembly on a glass substrate (Fig. 1a)^25^. A scanning electron microscopy (SEM) image (Fig. 1b) and an atomic force microscopy (AFM) image (Fig. 1c) show that the nanofibers have a rectangular cross section with a width and height of 300–700 and 50–200 nm, respectively. Our single-crystal X-ray diffraction measurements revealed that the nanofiber belongs to the monoclinic system with space group *P*2_1_/*n* and lattice parameters of *a* = 10.09(1) Å, *b* = 4.691(6) Å, *c* = 15.64(2) Å, *β* = 106.05(2)°, *V* = 712(2) Å^3^, and *Z* = 2 at *T* = 298 K, in agreement with the reported values for a single crystal of coronene at room temperature^26,27^. The long axis of the nanofiber is parallel to the stacking direction (// *b* axis).

A sample mounted in a liquid nitrogen flow cryostat with an optical window was cooled from room temperature under microscopic observation. When the temperature reached ~ 150 K, the nanofibers on the substrate started jumping violently (Supplementary Video 1). The nanofibers jumped continuously until the temperature reached 83 K, which is the lowest available temperature of the cryostat. After the temperature was held at 83 K for ~ 1 min, the nanofibers stopped jumping. The sample was then heated from 83 K. The nanofibers started jumping again at ~ 220 K and continuously jumped until room temperature. The sample stage of the cryostat has a diameter of 22 mm, and the optical window is located ~ 4 mm above the sample. After the cooling/heating cycle, the nanofibers had spread far outside of the stage and adhered to the window, indicating that the jumping distance and height of the nanofibers were on the order of centimeters.

**Figure 2:**
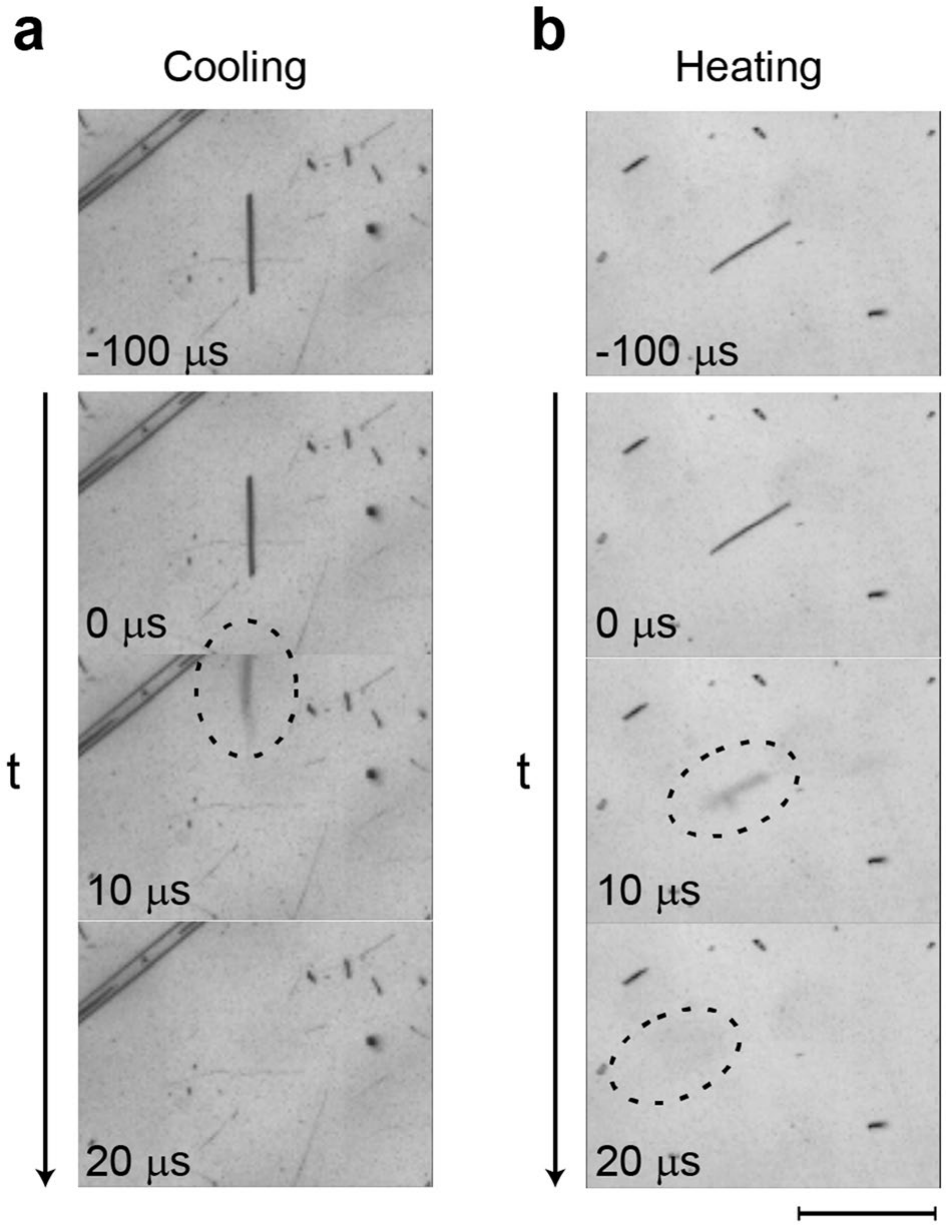
Jumping nanofibers recorded with high-speed camera operated at 10^5^ fps. **(a)** Snapshots recorded during cooling. (**b)** Snapshots recorded during heating. The topmost images were recorded 100 µs before jumping, showing that the jumps occurred without preliminary deformation. Scale bar: 100 µm.

To investigate the motion of the nanofibers at the moment of the jump, we recorded the nanofibers with a high-speed camera operated at 10^5^ fps (a time resolution of 10 µs). Figure 2a,b show typical snapshots of jumping nanofibers recorded during cooling and heating, respectively. We observed that the nanofibers tend to jump parallel to the substrate during cooling (Fig. 2a), whereas nearly perpendicular during heating (Fig. 2b). In addition, the images recorded well before jumping (*t* = − 100 µs) and just before jumping (*t* = 0 s) do not exhibit notable differences, indicating that the jump occurs suddenly without preliminary deformation or motion. The initial lateral velocity of the jumps during cooling was measured to be 10–20 m/s, while the vertical velocity during heating 1–5 m/s. These values are orders of magnitude faster than those previously reported for thermosalient crystals (typically ~ 0.5 m/s)^10^. Because of the high initial velocity, the jumping nanofibers are outside of the image area of 256 µm × 192 µm (or the focus depth of ~ 7 µm) within one or two frames (within 10 to 20 µs). This clearly shows that a higher time resolution is required to observe deformation of the nanofibers that causes the jump. This is in sharp contrast to the so far reported thermosalient effects, where the deformations of the crystals could be observed with a time resolution of ~ 1 ms^10,12,15^.

We attempted to capture deformation of the nanofibers by increasing the frame rate of the camera to its maximum value of 5.4 × 10^5^ fps (a time resolution of 1.85 µs). This is technically challenging, given the general characteristics of a high-speed camera. The frame rate and recording time of a high-speed camera are limited by the capacity of the on-camera memory and the writing speed to it. Because of these limitations, as the frame rate is increased, the active CCD area and the available recording time are decreased to compensate for the increased amount of data generated per unit time. The active area and recording time of our camera operated at 5.4 × 10^5^ fps are reduced to only 0.32 × 2.56 mm^2^ (16 × 128 pixels) and ~ 3 s, respectively (1.92 × 2.56 mm^2^ and ~ 6 s, respectively, at 10^5^ fps). Because of these restrictions, an elaborate recording procedure was required to shoot the jumps, which occur randomly in time and position (see “Methods”).

**Figure 3:**
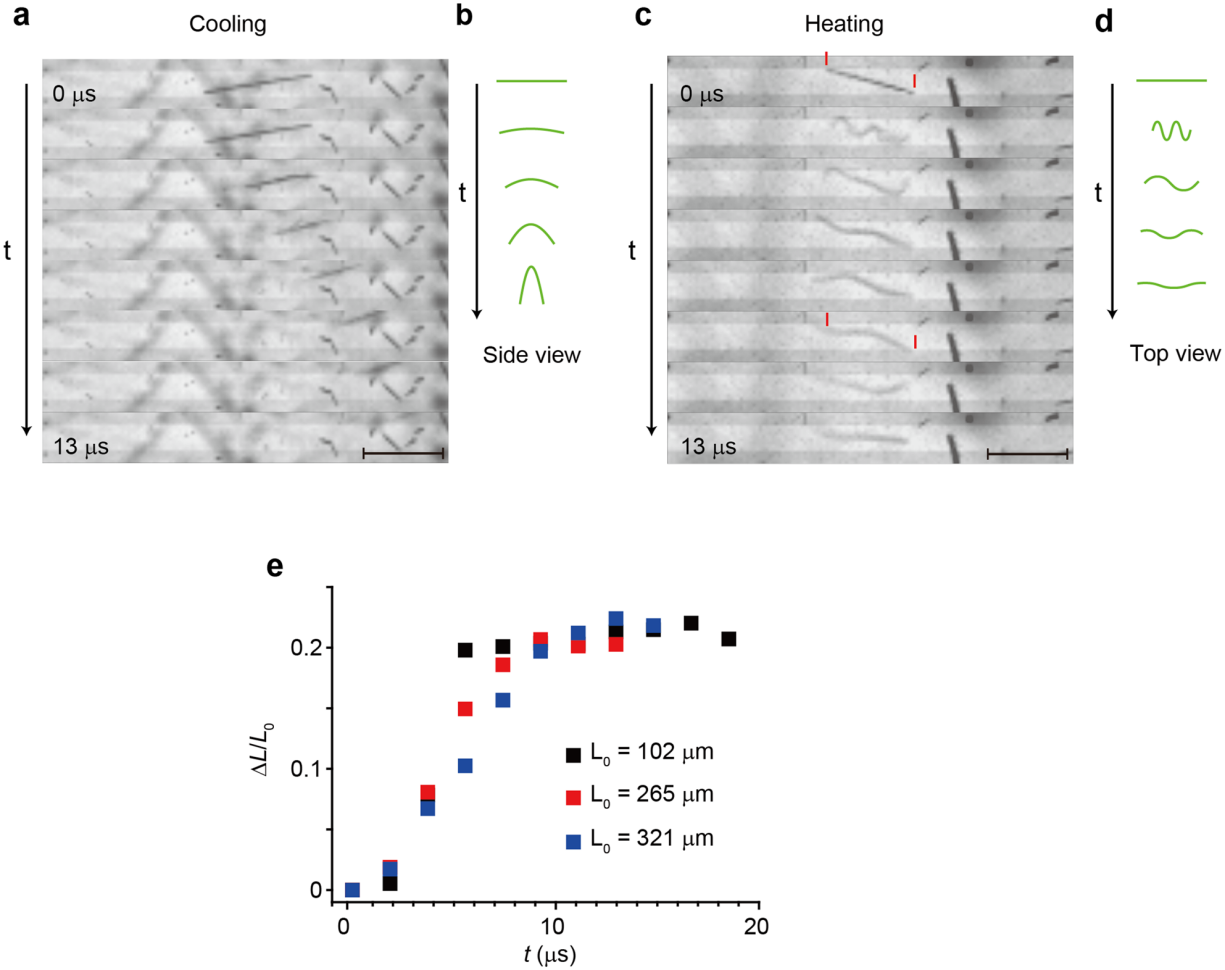
Jumping nanofibers recorded with high-speed camera operated at 5.4 × 10^5^ fps. **(a**) Snapshots recorded during cooling (scale bar: 100 µm). (**b)** Schematic illustrations of deformation of the nanofiber in (**a**). (**c)** Snapshots recorded during heating. Red bars indicate the initial length of the nanofiber. (**d)** Schematic illustrations of deformation of the nanofiber in (**c)**. (**e)** Elongation rates of nanofibers with different lengths as a function of time.

Figure 3a shows typical snapshots of a jumping nanofiber during cooling recorded at 5.4 × 10^5^ fps. The nanofiber suddenly bent nearly perpendicular to the surface with a large bending angle of ~ 180° (refer to Fig. 6c for the bending angle) and jumped along the direction of the nanofiber axis (Supplementary Video 2). We observed that the jumps during cooling were mostly initiated by such sudden sharp bending. Figure 3b schematically illustrates the deformation of the nanofiber at the moment of jumping. We also observed that approximately 20% of the jumps were accompanied by fracture. It has been reported that photosalient crystals having a needle-like shape with aspect ratios of up to ~ 5 showed cracking by the salient effect, while those of the same compound with aspect ratios of 20–40 showed a slight bending^28^. The large bending angle of the jumping nanofibers is in line with the reported results as they have very high aspect ratios of ~ 500.

Figure 3c shows typical snapshots of a jumping nanofiber during heating recorded at 5.4 × 10^5^ fps, exhibiting impressive behavior. The nanofiber wriggled like a worm or snake on a microsecond time scale before it lifted off the substrate. During the wriggling motion, the nanofiber was sharply bent with a large bending angle of  ~ 180° and a radius of curvature of a few micrometers. As the amplitude of the motion decreased, the nanofiber flew off in a direction nearly perpendicular to the substrate. Figure 3d schematically illustrates the deformation of the nanofiber. We observed that most of the jumps during heating were accompanied by the wriggling motion, although the number of waves over the length of the nanofiber varied depending on the fiber length (Supplementary video 3). It is rare that molecular crystals exhibit life-like motion in responding to external stimuli. Recently, Tong et al. reported that crystalline microwires of an organic compound under light irradiation show complex oscillatory motion with a frequency of ~ 1 Hz driven by photoisomerization^29^. The motion looks similar to the wriggling motion of the coronene nanofibers. However, the frequency of the wriggling motion is ~ 10^6^ Hz (= 1 µs^−1^), which is six orders of magnitude larger than that of the microwires, suggesting that the mechanisms behind these motions are very different.

The high-speed camera revealed another notable feature: the nanofibers were elongated during the wriggling motions. Eye guides for the initial length (red bars) in the first and sixth frames in Fig. 3c clearly show that the fiber length increased with time. Other snapshots that show the elongation are provided in Supplementary Information (Fig S1). The elongation rates, Δ*L*/*L*_0_ = (*L *− *L*_0_)/*L*_0_, were measured for several nanofibers with different lengths and are plotted as function of time in Fig. 3e (*L*: fiber length, *L*_0_: initial fiber length). The maximum elongation rate of all the measured nanofibers was found to be ~ 0.22 regardless of *L*_0_, and Δ*L*/*L*_0_ reached the maximum value within ~ 8 µs. On the other hand, the length change could not be observed for the nanofibers that jumped during cooling because of the sharp bending perpendicular to the substrate and the large lateral velocity.

**Figure 4:**
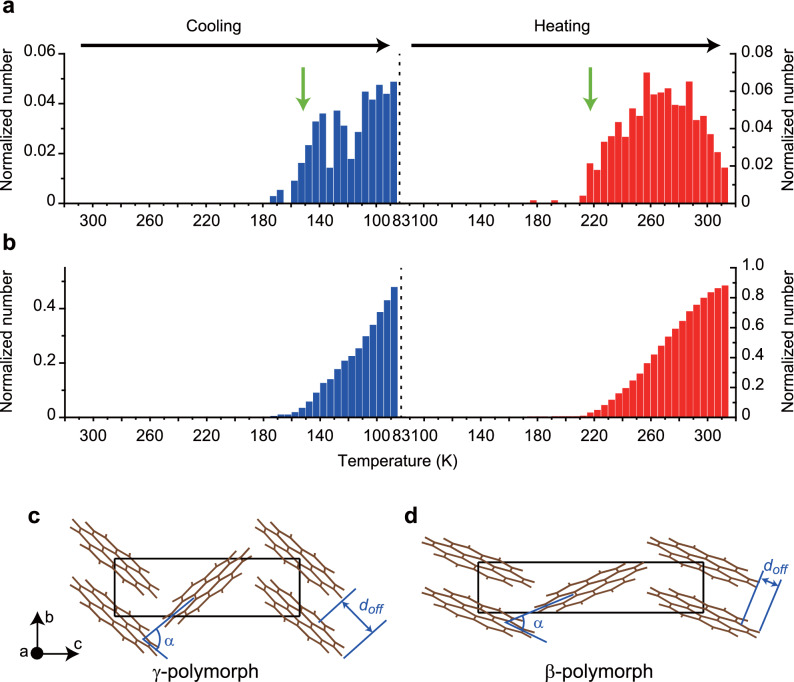
The number of times nanofibers jumped and the crystal structures of coronene. **(a)** The number of times nanofibers jumped as a function of temperature obtained by averaging over three measurements. Blue bars show the number during cooling and are normalized by the initial number of nanofibers in the image area. Red bars show the number during heating and are normalized by the number of nanofibers that jumped during cooling. Green arrows indicate the phase transition temperatures of coronene single crystals determined by the powder X-ray diffraction measurements^30^. (**b)** Histogram obtained by integrating that shown in (**a)**. (**c)** Structures of γ-polymorph. α: nearest-neighbor herringbone angle. *d*_*off*_: π-stacking offset. (**d)** Structure of β-polymorph.

To reveal the mechanism of the motions of the nanofibers, we counted jumps that occurred during a cooling/heating cycle of *T* = 300 → 83 → 300 K. Throughout the cycle, a video of the sample was recorded by a standard video camera (30 fps) installed on the microscope. In the image area (~ 4.2 mm in diameter), there were initially 300–500 nanofibers. The number of times of jumping was counted by visual inspection of the video and was accumulated for each 10 K temperature interval. We conducted this procedure for three samples, and the averaged values are shown in Fig. 4a. In the course of counting the jumps, we found the following two facts. First, no nanofibers jumped more than once during cooling. Second, the only nanofibers that had jumped during cooling jumped in the subsequent heating process. The histograms were normalized in light of these two facts (see the figure caption). Figure 4b shows a histogram obtained by integrating that shown in Fig. 4a. The histogram for cooling (blue bars) shows that ~ 50% of the nanofibers jumped by 83 K and that the count increases monotonically down to 83 K (note that no nanofibers jumped more than once during cooling). The monotonic increase suggests that further cooling results in more jumps. The histogram for heating (red bars) shows that ~ 90% of the nanofibers that experienced jump during cooling jumped again by 300 K, and thus it shows a saturation behavior around 300 K.

Recently, Potticary et al. discovered a new polymorph of coronene crystals, which can be obtained by recrystallization under a magnetic field^30,31^. Whereas a coronene crystal grown under ambient conditions has a γ-herringbone structure (Fig. 4c), the new polymorph has a β-herringbone structure that is characterized by a smaller nearest-neighbor herringbone angle (α = 49.71° and 95.86° in the γ- and β-polymorphs, respectively), and a smaller π-stacking offset of coronene disks (*d*_*off*_ = 3.84 and 1.606 Å in the γ- and β-polymorphs, respectively) (Fig. 4d). Moreover, they found that the γ ↔ β phase transition can be induced by varying the temperature^30,31^. They performed powder X-ray diffraction measurements of single coronene crystals during a cooling/heating cycle of *T* = 300 → 12 → 300 K and found that the diffraction peaks originated from the β-polymorph appeared at ~ 150 K during cooling and disappeared at ~ 220 K during heating.

The temperatures at which the β-polymorph appears and disappears, as determined by the powder X-ray diffraction measurements, are indicated by green arrows in Fig. 4a. These temperatures are in good agreement with those at which the nanofibers started jumping, suggesting that the jumps are related to the γ ↔ β phase transitions. The crystal structure of the β-polymorph is also monoclinic with space group *P*2_1_/*n* and lattice parameters of *a* = 10.386(1) Å, *b* = 3.8212(3) Å, *c* = 17.211(2) Å, β = 96.24(1)°, *V* = 679.0(1) Å^3^, and *Z* = 2 at *T* = 80 K^30,31^. The *b* value of the γ-polymorph is 23% larger ([4.691 – 3.8212 Å]/3.8212 Å) = 0.23) than that of β-polymorph. This value agrees well with the elongation rate of jumping nanofibers during heating (~ 22%). This strongly supports that the jumps are attributed to the γ ↔ β phase transition.

**Figure 5:**
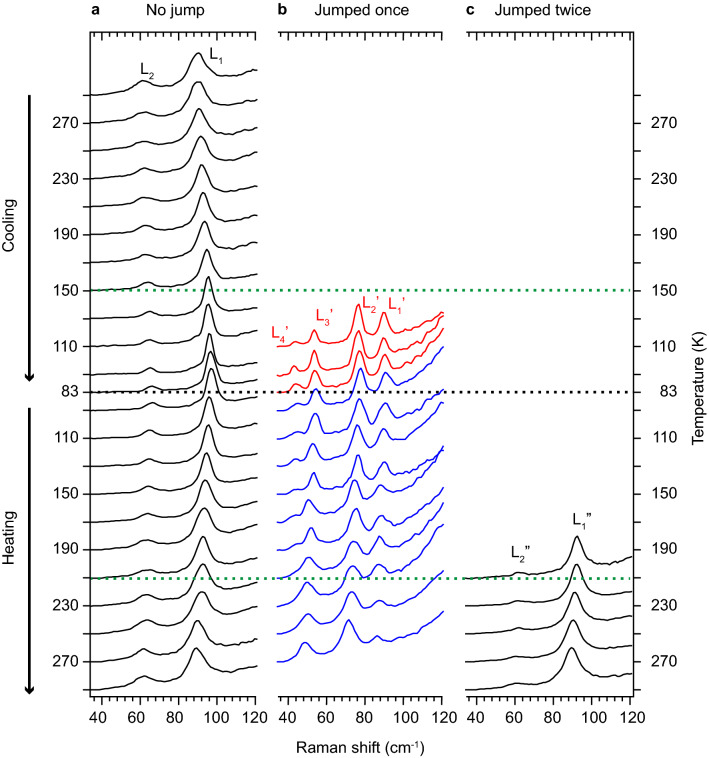
Raman spectra of single coronene nanofibers measured during the cooling/heating cycle of 300→83→300 K. (**a)** Spectra of nanofibers that did not jump throughout the cooling/heating cycle. (**b)** Red curves: Spectra of nanofibers that jumped during cooling measured immediately after landing on the substrate. Blue curves: spectra of nanofibers that jumped during cooling but did not jump during heating. (**c)** Spectra of nanofibers that jumped during heating measured immediately after landing on the substrate.

To further confirm the relationship between the jump and the phase transitions, we performed Raman microscopy on single nanofibers in the lattice vibration frequency region (40–120 cm^−1^). As shown in Fig. 4b, ~ 50% of the nanofibers did not jump throughout the cooling/heating cycle. Figure 5a shows the Raman spectra of such nanofibers that did not jump. At room temperature (topmost spectrum), the spectrum shows two peaks at ~ 90 and ~ 60 cm^−1^ (labeled L_1_ and L_2_, respectively) that are due to lattice vibrations of the γ-polymorph. Although another peak at ~ 40 cm^−1^ has been reported for a single crystal of coronene at room temperature^32^, we could not observe it because of the filter cutoff frequency of our setup. We also performed polarized Raman microscopy on single nanofibers and assigned these peaks to libration modes, which are torsional vibrations around the molecular axes (Fig. S2 and Table S1). As the temperature is decreased, these peaks shifted to higher frequencies, reflecting contraction of the lattice. However, the major spectral features did not change even at *T* < 150 K, suggesting that the nanofibers remained in γ-polymorph. During heating from 83 K, the peaks shifted to lower frequencies owing to lattice expansion.

During cooling from *T* = 150 → 83 K, ~ 50% of the nanofibers jumped, as shown in Fig. 4b. In this temperature range, the Raman spectra of the nanofibers that jumped were measured immediately after they landed on the substrate (red curves in Fig. 5b). The spectra are clearly different from those of the γ-polymorph, indicating that the nanofibers were transformed into the β-polymorph. We performed the Raman measurements on ~ 50 nanofibers that jumped during *T* = 150 → 83 K and confirmed that they were all transformed into the β-polymorph. Subsequent heating of these nanofibers from 83 K did not result in major spectral changes unless they jumped (blue curves in Fig. 5b). The spectra of the β-polymorph show four peaks at 93, 80, 57, and 46 cm^−1^ (labeled L_1_′, L_2_′, L_3_′, and L_4_′, respectively, in Fig. 5b). The polarized Raman spectra showed that these peaks could not be consistently assigned to libration modes (Fig. S2 and Table S1). This suggests that the librations are strongly influenced by the phase transition and are not well-defined in the β-polymorph. A recently study on thermosalient crystals of perhydropyrene, which is a polycyclic hydrocarbon similarly to coronene, showed that the librations play key roles in the thermosalient phase transition^33^. Therefore, it is suggested that the librations are also critically involved in the phase transition of the coronene nanofibers. A further study on the lattice vibrations in the β-polymorph is needed.

During heating from *T* = 220 → 300 K, ~ 90% of the nanofibers that jumped during cooling jumped again, as shown in Fig. 4b. The spectra of the nanofibers that jumped at *T* > 220 K were measured immediately after landing on the substrate (Fig. 5c). The spectra changed again, and two peaks labeled L_1_″ and L_2_″ were observed at the same positions as L_1_ and L_2_, showing that the phase transition occurred with the jump and that the nanofibers were transformed back to the γ-polymorph. We performed the Raman measurements on ~ 30 nanofibers that jumped during *T* = 220 → 300 K and observed that all of them underwent the phase transition. On the other hand, ~ 10% of the nanofibers of the β-polymorph did not jump up to *T* = 300 K and retain the β form. The presence of the β-polymorph at room temperature after the cooling/heating cycle was also confirmed by the powder X-ray diffraction measurements (Figs. S3, S4, and S5). It is notable that the jumping occurred over the wide temperature ranges both during cooling (*T* = 150–83 K) and heating (*T* = 220–300 K). Namely, the temperature at which the jumping occurred was largely different for each nanofiber. Although our microscopic and spectroscopic investigations could not identify the factors that lead to the different jumping temperature, it may be attributed to the intrinsic factors, such as structural defects and strain, and the extrinsic factors, such as thermal contact between the substrate.

Naumov et al. have systematically investigated the phase transitions in several thermosalient crystals and found that they have common characteristics^10^. Namely, the crystal symmetry is preserved and the lattice undergoes anisotropic distortion through the transition. These characteristics indicate that the transition is a diffusionless transformation with small cooperative movement of individual molecules. Therefore, they claimed that the phase transition in the thermosalient crystals is an organic analogues of a martensitic transition in inorganic materials^10,12^. The phase transitions in the coronene nanofibers also have these characteristics. The crystal symmetry (*P*2_1_/*n*) is preserved and the unit cell undergoes the anisotropic distortion: it expands (shrinks) along the *a* and *c* axes and shrinks (expands) along the *b* axis in the γ → β (β → γ) transition (Fig. 4c,d). Consequently, the phase transition of the coronene nanofibers is also considered to be martensitic. A martensitic transition proceeds by a movement of the habit plane (the plane between the parent and product phases). Naumov et al. indeed observed the movement of the habit plane across the thermosalient crystals at the moment of the phase transition and measured its velocity to be ~ 0.5 m/s^10,12^. We observed that the phase transition over entire coronene nanofibers with lengths of up to ~ 320 µm were completed within 8 µs (Fig. 3e). Assuming that the transition proceeds from one end of the nanofiber to the other, the velocity of the habit plane moving across the lengths of nanofibers is estimated to be ~ 40 m/s, which is two orders of magnitude faster than those for the thermosalient crystals. This significantly fast movement of the habit plane generates an abrupt internal force along the long axis, which plays a key role in the motions of the nanofibers, as is described in the following sections.

The most impressive feature in the jumps of the nanofibers is the fast wriggling motion as they jump during heating. Our next focus is to elucidate the mechanism that cause the motion. We observed that the nanofibers rapidly increased their length as they started wriggling. This indicates that just before the wriggling motion started (*t* = 0), a compressive force was generated along the nanofiber axis. Instead of considering this force to be internally generated, we simply treat it as an effective compressive force that is applied externally. This allows us borrowing the knowledge from structural mechanics: a slender elastic body, such as a beam or a column, subjected to an external compressive force along the long axis exhibits *dynamical (buckling) instability* that can lead to their sudden and large deformation when the force exceeds a critical value. We analyze the motion of the nanofibers in terms of the dynamical instability.

**Figure 6:**
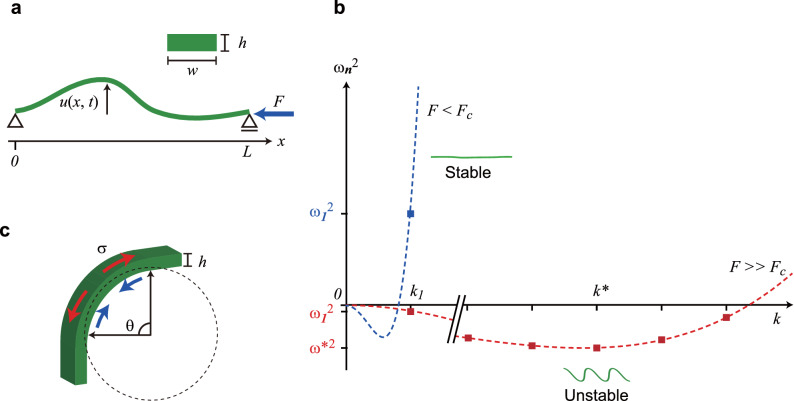
Theoretical analysis on deformation of a nanofiber. **(a)** The model used in the linear stability analysis. The inset shows the cross section of the nanofiber. (**b)** Schematic plots of Eq. (4). (**c**) Schematic representation of stresses in a bent slender elastic body. Red arrows represent the tensile stress generated in the outer portion, and blue arrows the compressive stress generated in the inner portion.

Figure 6a shows the model used in our analysis. A nanofiber of length *L* has a rectangular cross section with a width *w* and height *h*. Both ends of the nanofiber are assumed to be free to pivot, yielding the boundary conditions *u*(0, *t*) = *u*(*L*, *t*) = 0, where *u*(*x*, *t*) is the displacement at position *x*. The equation of motion of this nanofiber under the compressive force *F* is written as

1 $${\mathrm{\rho}} {A}\frac{{\partial }^{2}u}{\partial {t}^{2}}=-D\frac{{\partial }^{4}u}{\partial {x}^{4}}-F\frac{{\partial }^{2}u}{\partial {x}^{2}},$$

2 $$D=IE=\frac{w{h}^{3}}{12}E,$$

where *ρ* is the density, *A* = *wh* is the cross-sectional area, *I* is the moment of inertia of the area, and *E* is Young’s modulus^34^. We conducted a linear stability analysis of the nanofiber based on Eqs. (1) and (2)^34,35^. The solution of Eq. (1) can be written as

3 $$u\left(x, t\right) exp\left[i\left({k}_{n}x-{\omega }_{n}t\right)\right],$$

where *k*_*n*_ is the wavenumber and is given by *k*_*n*_ = *nπ*/*L* (*n* = 1, 2, …). Then, the following dispersion relation is obtained from Eq. (1):

4 $$-\rho A{{\omega }_{n}}^{2}=-D{{k}_{n}}^{4}+F{{k}_{n}}^{2}.$$

Schematic plots of Eq. (4) are shown in Fig. 6b. When *F* < *F*_*c*_ (blue dashed curve), ω_*n*_^2^ > 0 for any *k*_*n*_, where *F*_*c*_ is the *critical Euler buckling force* that is given by *D*(*π*/*L*)^2^. In this force regime, ω_*n*_ is a real number, and thus *u*(*x*, *t*) has the form of a propagating wave, indicating that the motion of the nanofiber is stable. By contrast, when *F* >  > *F*_*c*_,* ω*_*n*_^2^ < 0 for a certain range of *k*_*n*_ (red dashed curve). Within this range, ω_*n*_ is an imaginary number, and thus the amplitude of *u*(*x*, *t*) diverges with time once infinitesimally small displacement occurs, implying that the nanofiber is dynamically unstable. The *k*_*n*_ at which *ω*_*n*_^2^ has its minimum value is obtained as *k*_n_* = (*F*/2*D*)^1/2^ by finding the minimum *ω*_*n*_^2^ from Eq. (4) under the assumption that *k* is continuous. The mode defined by *k*_*n*_ = *k*_n_* is most unstable and rapidly growing. Therefore, when the force in this regime is abruptly generated by the phase transition, the nanofiber is transformed into a wave-like form with λ* = 2π/*k*_n_*. We consider that the wriggling motion is a manifestation of this instability characterized with λ*.

Next, we show that our model also can quantitatively capture the key features of the wriggling motion. We evaluated the force *F* generated by the phase transition, which is given by *F* = 8π^2^*D*/*λ*^2^ = 2π^2^*wh*^3^*E*/3*λ*^2^. The second snapshot in Fig. 3b (*t* = 1.85 µs) shows that the nanofiber (*L* = 100 µm) has *n* = 4, yielding *λ* = 2*L*/*n* = 50 µm. Using *E* = 1.1 × 10^9^ N/m^2^ ^36^, and the typical values of *w* = 500 nm and *h* = 200 nm, *F* was calculated to be 1.16 × 10^–8^ N (~ 0.12 MPa). Further using the *F* value and *ρ* = 1.38 × 10^3^ kg/m^3^ ^36^, the characteristic time of the waveform growth, which is given by *τ* = 1/ω= [(*Dk*^4^ − *Fk*^2^)/*ρA*]^−1/2^, was obtained to be *τ* = 1.23 µs. The snapshots show that the *n* = 4 wave-like shape developed at *t* = 1.85 µs, which is in reasonably good agreement with the evaluated *τ*. The validity of our model was also confirmed for observed wriggling motions of other nanofibers. We concluded that the origin of the motion is the dynamical instability induced by the phase transition.

Because of the sharp bending and large initial velocity, the change in the length could not be observed for jumping nanofibers during cooling. However, it is most probable that the nanofibers shortened at the moment of jumping, as opposed to the elongation observed during heating, and thus the tensile force was generated along the long axis. The tensile force does not cause the instability. This is consistent with the fact that no wriggling motion was observed for the nanofibers that jumped during cooling. The sharp bending at the moment of the jump is probably attributed to exfoliation of nanofibers from the surface of the substrate. The nanofibers were prepared by solution evaporation on the surface (see “Methods”) and, therefore, adhered to the surface. As the nanofibers were shortened by the phase transition, they were exfoliated from the surface. When exfoliation proceeds from one end of a nanofiber to the other, it can be bent perpendicular to the surface and jump from the surface.

We observed that the jumping nanofibers were sharply bent with a bending angle of up to ~ 180° and a radius of curvature of a few micrometers without breakage. This extraordinary flexibility of the nanofibers make the γ ↔ β phase transition to be the reversible nanofiber-to-nanofiber transition. For a complete understanding of the origin of the highly flexible nature, the mechanical properties of the nanofibers are essential information. The mechanical properties of organic crystals, such as flexibility, elasticity, and hardness, have been investigated by various techniques and discussed in relation to the crystal structure and molecular interactions^37–41^. However, a challenge to apply such measurements to the nanofibers is out of the scope of the current study. Instead, here we simply note that the size and shape are critical to the high flexibility. When a slender elastic body is bent, the outer portion undergoes tensile stress, whereas the inner portion undergoes compressive stress (Fig. 6c)^42^. The tensile stress at the outer surface (*σ*) is proportional to the bending angle *θ* and inversely proportional to the thickness *h* (the Hooke’s law) (Fig. 6c). When *σ* exceeds a critical value, flexural failure initiated by rupture of the outer surface occurs^43^. Owing to the nanoscale *h*, *σ* in a bent nanofiber is small even at the large *θ*, leading to flexibility far superior to bulk crystals, which often splinter through thermosalient effects due to the lack in flexibility. This discussion implies that the size and shape, the elastic properties, and the force generated by the phase transition together lead to the ultrafast, large deformation of the nanofibers without breakage. Thus our results demonstrate that crystals with controlled size and shape have the potential to exhibit unrevealed phase-transition-induced motions, which cannot emerge in bulk crystals. This concept may provide a new insight into the design and fabrication of crystal-based high-speed mechanical devices with large displacements.

## Methods

### Nanofiber fabrication

The fabrication process of coronene nanofibers was described elsewhere^25^. Briefly, the sample solution (~ 3.0 mM) was prepared by dissolving coronene in chloroform by sonication. Approximately 10 µL of the sample solution was dropped onto a substrate (an untreated microscope cover glass), and the substrate was immediately placed in a glass container (a Petri dish with a lid), which was filled with chloroform vapor by depositing a few drops of chloroform at the bottom. After a few hours, during which the solvent was evaporated, the sample was extracted from the container and dried under ambient conditions.

### SEM and AFM

SEM images were obtained using a scanning electron microscope (JEOL, JSM-7000F). The sample was carbon-coated to avoid charging. The topography of the sample was observed using AFM (Veeco, Caliber) in tapping mode.

### High-speed camera recording

An epi-illumination microscope (Olympus, BX-51) equipped with a motorized stage (Prior, H-101) and a liquid nitrogen flow optical cryostat (Linkam) was used. The sample was under a nitrogen gas atmosphere. High-speed videos were recorded using a high-speed camera (Photron, FASTCAM) mounted on the microscope. A video of a selected nanofiber was loop-recorded to on-camera memory while the sample temperature was varied at 5–10 K/min. When the nanofiber jumped, the loop recording was immediately stopped manually so that the last ~ 3 s of the video were saved.

### Raman microscopy on single nanofibers

The microscope and cryostat used for high-speed camera recording were also used for the Raman microscopy. The output of a continuous-wave diode laser (*λ* = 785 nm) was coupled to the microscope, directed toward the sample by a dichroic mirror (Semrock), and focused onto the sample by a 50 × IR objective lens (Olympus)^44^. Scattered light from the sample was collected by the same objective lens and imaged on the entrance slits of an imaging monochromator (Acton Research, SpectraPro 2150) through a long-pass filter (Semrock) that blocked the excitation laser. The spectra were recorded by a liquid-nitrogen-cooled back-illuminated CCD camera (Princeton Instruments, Spec10, 1340 pixels × 400 pixels) attached to the monochromator. For the polarized Raman microscopy measurements, polarizers were placed before and after the sample.

### Single-crystal X-ray diffraction experiments

A nanofiber was picked up from the substrate with a wooden stick and attached to the tip of a glass capillary using epoxy. The single-crystal X-ray diffraction experiments were performed on a CCD-type diffractometer (Bruker SMART APEX II) with graphite-monochromated Mo Kα radiation (*λ* = 0.71073 Å) at 298 K.

## Supplementary Information

Supplementary Information 1.

Supplementary Video 1.

Supplementary Video 2.

Supplementary Video 3.

## Data Availability

The data that support the findings of this study are available from the corresponding author upon reasonable request.
