# Supplementary material for: Phase-transition-induced jumping, bending, and wriggling of single crystal nanofibers of coronene
Source: Sci Rep. 2021 Feb 4;11:3175. doi: 10.1038/s41598-021-82703-5 (PMC7862269; doi:10.1038/s41598-021-82703-5)
Supplement: Supplementary file 1 — Supplementary Information 1. [file 41598_2021_82703_MOESM1_ESM.pdf]

Supplementary Information for

# Phase-transition-induced jumping, bending, and wriggling of single crystal nanofibers of coronene

Ken Takazawa*[1], Jun-ichi Inoue[2], Kazutaka Mitsuishi[3], Yukihiro Yoshida[4, 5], Hideo Kishida[6], Paul Tinnemans[7], Hans Engelkamp[8], and Peter. C. M. Christianen[8]

[1]*Center for Green Research on Energy and Environmental Materials, National Institute for Materials Science, Tsukuba, Ibaraki 305-0003, Japan*

[2]*MANA, National Institute for Materials Science, Tsukuba, Ibaraki 305-0044, Japan*

[3]*Research Center for Advanced Measurement and Characterization, National Institute for Materials Science, Tsukuba, Ibaraki 305-0047, Japan*

[4]*Division of Chemistry, Graduate School of Science, Kyoto University, Kitashirakawa-Oiwakecho, Sakyo-ku, Kyoto 606-8502, Japan*

[5]*Faculty of Agriculture, Meijo University, Tempaku-ku, Nagoya 468-8502, Japan*

[6]*Department of Applied Physics, Nagoya University, Chikusa, Nagoya 464-8603, Japan*

[7]*Department of Solid State Chemistry, Radboud University, 6500 GL Nijmegen, The Netherlands*

[8]*High Field Magnet Laboratory (HFML-EMFL), Radboud University, 6525 ED Nijmegen, The Netherlands*

**Supplementary note 1: The elongation of jumping nanofibers during heating**

Fig. S1a and b show snapshots of jumping nanofibers during heating recorded at 5.4 $\times 10^5$ fps. Each image is shifted horizontally so that the right tips of the nanofibers are aligned. The increases in the lengths are clearly seen.

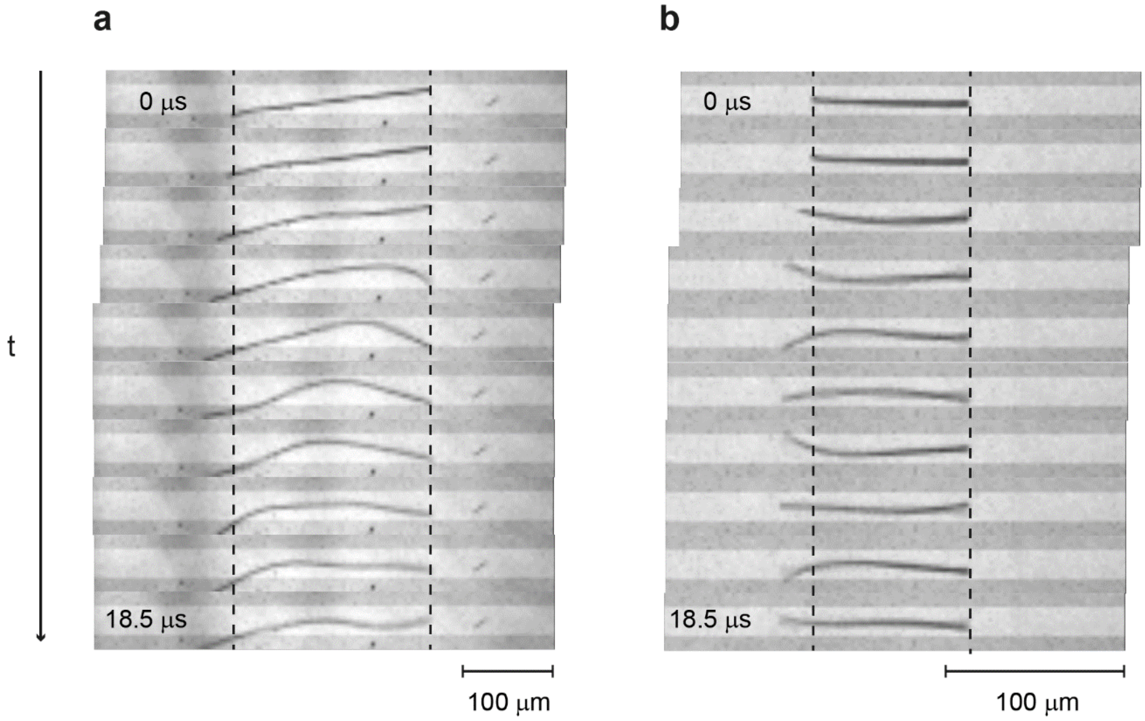


**Fig. S1 Snapshots of jumping nanofibers during heating. a,** The initial lengths: 265 μm. **b,** The initial length: 102 μm. The dashed lines are eye guides for the initial lengths.

**Supplementary note 2: The lattice vibrations of γ- and β-nanofibers of coronene**

We measured the polarized Raman spectra of single γ- and β-nanofibers in the frequency region of 40–120 $cm^{-1}$ to investigate the lattice vibrations. The polarized Raman spectra of the γ-nanofiber measured at T = 290 K are shown in the left panels of Fig. S2. The polarizations are shown in the figure; for example, (||*b*, ⊥*b*) indicates that the excitation and detection are polarized parallel and perpendicular to the *b*-axis, respectively. Two peaks at 61 and 91 $cm^{-1}$ were observed in the (||*b*, ||*b*) and (⊥*b*, ⊥*b*) spectra, and two peaks at 64 and 87 $cm^{-1}$ were observed in the (||*b*, ⊥*b*) and (⊥*b*, ||*b*) spectra. Although another peak at ~40 $cm^{-1}$ was reported in the unpolarized Raman spectrum of single crystals of coronene at room temperature[1], we could not observed it because of the filter cutoff of our setup. The polarized Raman spectra of the β-nanofiber measured at *T* = 110 K are shown in the right panels of Fig. S2. Three peaks at 46, 57, 80 $cm^{-1}$ were observed in the (||*b*, ||*b*) and (⊥*b*, ⊥*b*) spectra, and two peaks at 78 and 93 $cm^{-1}$ were observed in the (||*b*, ⊥*b*) and (⊥*b*, ||*b*) spectra.

Both the γ- and β-crystals of coronene belong to the monoclinic system of the space group $P2_{1/}n$ with two molecules in the unit cell. In these crystals, there are six Raman active lattice modes, which are classified into the three $A_g$ species and the three $B_g$ species of the factor group $C_{2h}$.[2] For many polyaromatic hydrocarbon (PAH) crystals, their lattice vibrations can be approximated by librations[2–4]. The librations are torsional vibrations (oscillations) around the three principal axes of inertia of each molecule. In the coronene crystals, since there are two molecules in the unit cell, three libration modes, which result from the librational motions around the three principal axes of each molecule, further split into two modes, namely, the $A_g$ and $B_g$ modes.

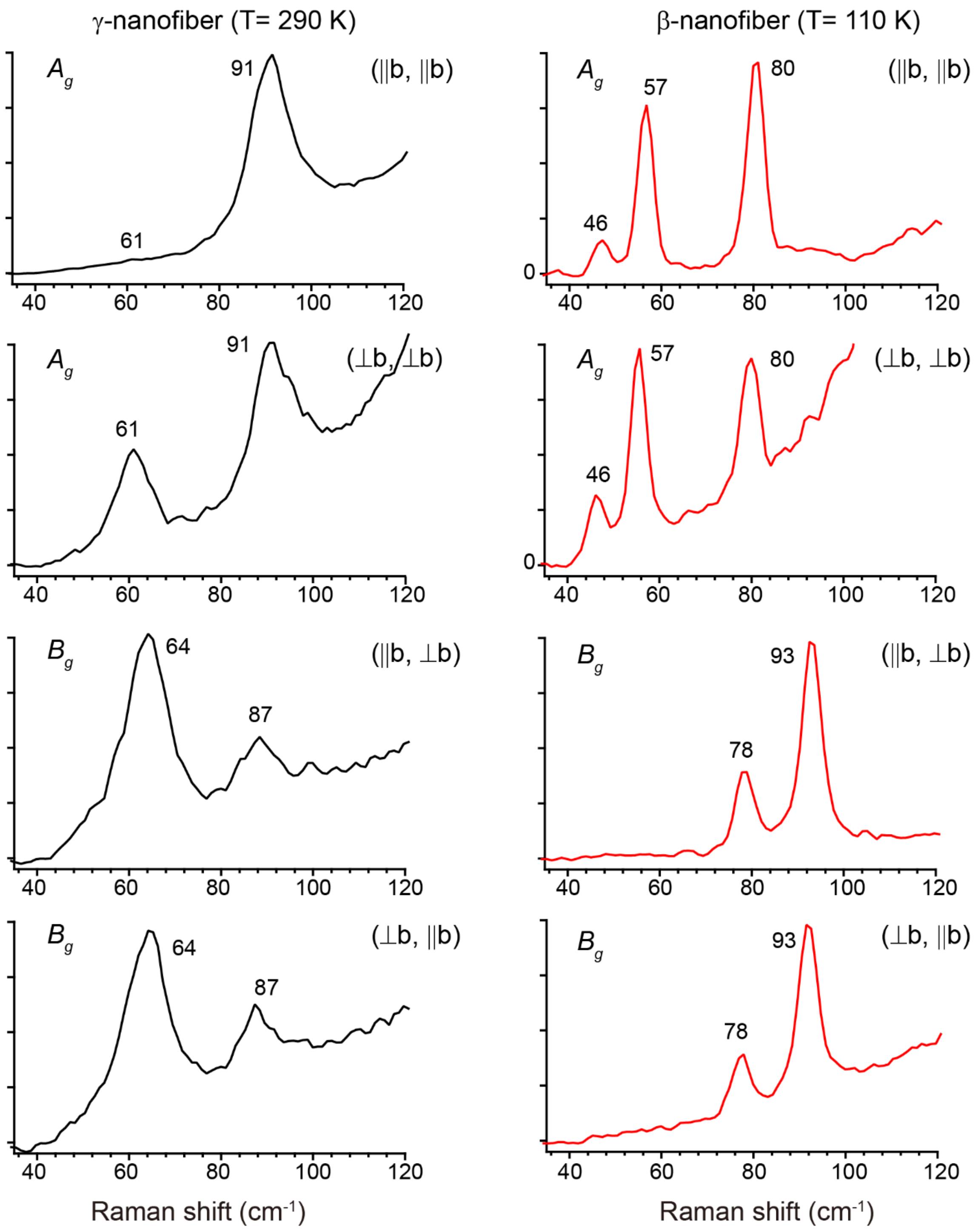


**Fig. S2 Polarized Raman spectra of single γ- and β-nanofibers.** The left panels: γ-nanofiber. The right panels: β-nanofiber.

The $A_g$ mode results from the in-phase coupling of the librations of the two molecules around the same principal axis and, the $B_g$ mode results from the out-of-phase coupling. The splitting frequencies between the $A_g$ and $B_g$ modes for the previously studied PAH are zero to a few wavenumbers[2–4]. In the polarized Raman spectra, the $A_g$ modes appear only in the (||*a*, ||*a*), (||*b*, ||*b*), and (||*c*, *c*||) spectra, while the $B_g$ modes appear in the (||*a*, ||*b*) and (||*b*, ||*c*) spectra.

On the basis of the selection rules, the peaks observed in the (||*b*, ||*b*) and (⊥*b*, ⊥*b*) spectra of the γ-nanofiber are assigned to the $A_g$ modes, and those in the (||*b*, ⊥*b*) and (⊥*b*, ||*b*) spectra are assigned to the $B_g$ modes. The frequencies of the observed modes are listed in Table S1. These modes can be divided into two pairs of the $A_g$ and $B_g$ modes (one at ~60 $cm^{-1}$ and the other at ~90 $cm^{-1}$), and the frequency differences between the $A_g$ and $B_g$ modes in each pair are a few wavenumbers. Thus, theses peaks can be assigned to the libration modes around the two principal axes of inertia. The other $A_g$ and $B_g$ pair probably locates at ~40 $cm^{-1}$, where the peak was observed in the unpolarized Raman spectrum[1].

The peaks observed in the (||*b*, ||*b*) and (⊥*b*, ⊥*b*,) spectra of the β-nanofiber are assigned to the $A_g$ modes, and those observed in the (||*b*, ⊥*b*) and (⊥*b*, ||*b*) spectra are assigned to the $B_g$ modes. The frequencies of the observed modes are listed in Table S1. Unlike the γ-nanofiber, the observed modes cannot be simply divided into the $A_g$ and $B_g$ pairs, even assuming that there is another $B_g$ mode below the filter cutoff frequency (< 40 $cm^{-1}$). This may suggest that the libration model is not appropriate for the β-crystal. The assignment of these modes needs further investigation.

**Table S1. Lattice vibrations of γ- and β-nanofibers of coronene**

| γ-nanofiber | |
|---|---|
| (40 $cm^{-1}$)[1)] | ($A_g$, $B_g$) |
| 61 | $A_g$ |
| 64 | $B_g$ |
| 87 | $B_g$ |
| 91 | $A_g$ |
| **β-nanofiber** | |
| 46 $cm^{-1}$ | $A_g$ |
| 57 | $\mathrm{A_g}$ |
| 78 | $B_g$ |
| 80 | $A_g$ |
| 93 | $B_g$ |

1) Not observed in this study. The peak at ~40 $cm^{-1}$ was reported in Ref. [1].

**Supplementary note 3: The Powder X-ray measurements of coronene nanofibers**

First, we performed the powder X-ray diffraction measurements of coronene nanofibers at room temperature (black curve in Fig. S3). The diffraction pattern agreed with that of single crystals of the γ-polymorph[1]. Then, the sample was submerged in liquid nitrogen. After this temperature treatment, the X-ray diffraction of the sample was measured at room temperature. After the temperature treatment of 15 min, the diffraction pattern did not change compared to that obtained without the temperature treatment (red curve in Fig. S3). However, after the temperature treatment of 2 days, the diffraction peaks due to the β-polymorph, e.g. the two peaks at ~10 degrees, were observed (blue curve in Fig. S4). The analysis of the diffraction pattern showed that the sample contained approximately ~6% of the β-polymorph (Fig. S5).

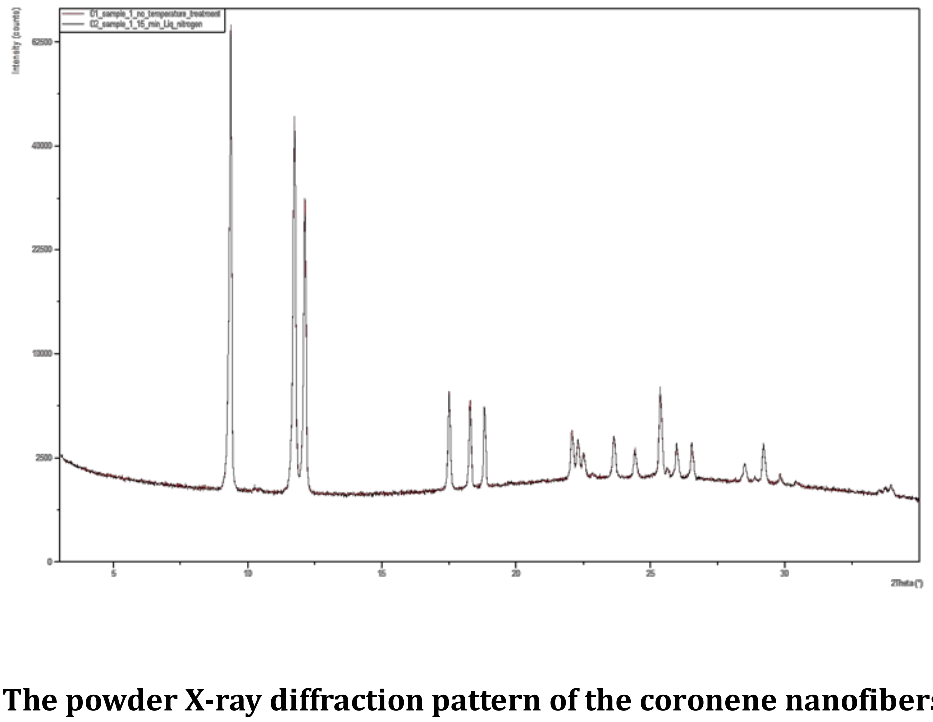


**Fig. S3 The powder X-ray diffraction pattern of the coronene nanofibers at room temperature.** Red curve: The sample without the temperature treatment. Black curve: The sample after the temperature treatment of 15 min.

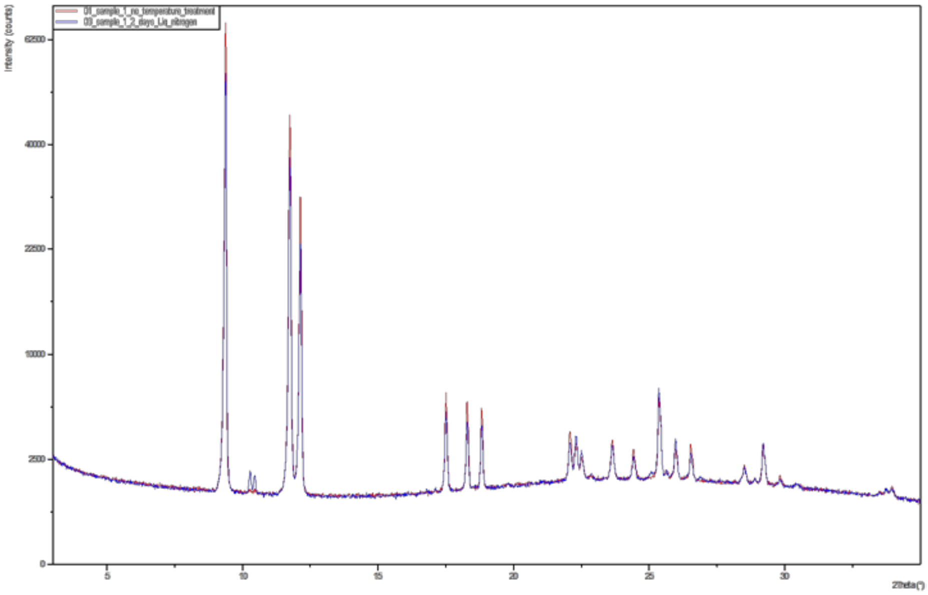


**Fig. S4 The powder X-ray diffraction pattern of the coronene nanofibers at room temperature.** Red curve: The sample without temperature treatment. Red curve: The sample after the temperature treatment of 2 days.

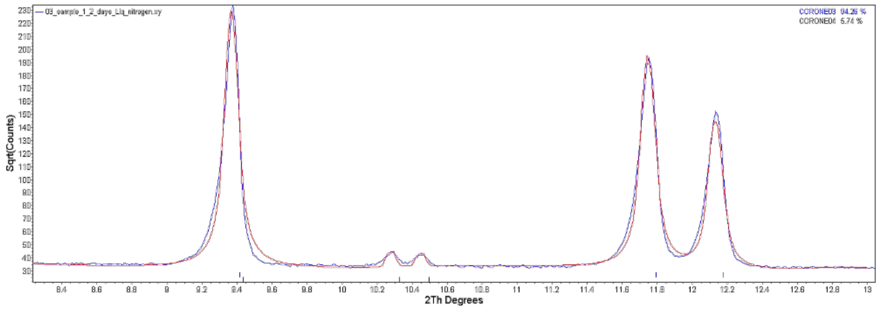


**Fig. S5 The experimental and simulated diffraction patterns of the sample after the temperature treatment of 2 days.** Blue curve: The experimental curve. Red curve: The simulated curve obtained by assuming that the sample contains ~6% of the β-polymorph.

## References


1. Ohno, K., Kajiwara, T., & Inokuchi, H. Vibrational analysis of electronic transition bands of coronene. *Bull. Chem. Soc. Jpn.* **45**, 996-1004 (1972).
2. Suzuki, M., Yokoyama, T., & Ito, M. Polarized Raman spectra of naphthalene and anthracene single crystals. *Spectrochimica Acta.* **A24**, 1091-1107 (1968).
3. Fruhling, A. Low Frequency Raman Spectrum of a Benzene Single Crystal. *J. Chem. Phys.* **18**, 1119-1119 (1950).
4. Ito, M., & Shigeoka, T. Raman spectra of benzene and benzene-d6 crystals. *Spectrochimica Acta*, **22**, 1029-1044 (1966).
5. Potticary, J. et al. An unforeseen polymorph of coronene by the application of magnetic fields during crystal growth. *Nat. Commun.* **7**, 11555 (2016).
